# Genome Scan Analysis for Advancing Knowledge and Conservation Strategies of Primitivo Clones (*Vitis vinifera* L.)

**DOI:** 10.3390/plants14030437

**Published:** 2025-02-02

**Authors:** Silvia Procino, Monica Marilena Miazzi, Vito Nicola Savino, Pierfederico La Notte, Pasquale Venerito, Nunzio D’Agostino, Francesca Taranto, Cinzia Montemurro

**Affiliations:** 1 Institute of Biosciences and Bioresources (CNR-IBBR), 70126 Bari, Italy; silviaprocino@cnr.it; 2 Department of Soil, Plant and Food Sciences, University of Bari Aldo Moro, 70126 Bari, Italy; monicamarilena.miazzi@uniba.it (M.M.M.); cinzia.montemurro@uniba.it (C.M.); 3 Fondazione ITS Agroalimentare Puglia, 70010 Locorotondo, Italy; viton.savino@gmail.com; 4 Support Unit Bari, Institute for Sustainable Plant Protection, National Research Council of Italy (CNR), 70126 Bari, Italy; pierfederico.lanotte@ipsp.cnr.it; 5 Centro di Ricerca, Sperimentazione e Formazione in Agricoltura “Basile Caramia” (CRSFA), 70010 Locorotondo, Italy; pasqualevenerito@crsfa.it; 6 Department of Agricultural Sciences, University of Naples Federico II, 80055 Portici, Italy; nunzio.dagostino@unina.it; 7 SINAGRI S.r.l., Spin-Off of the University of Bari Aldo Moro, 70126 Bari, Italy

**Keywords:** genotyping by sequencing, single nucleotide polymorphisms, outlier SNPs, molecular markers, genetic divergence, viticultural zoning

## Abstract

The success of the Primitivo variety underscores the critical need for the managing of clone genetic conservation, utilization, and improvement. By combining genomic and environmental data, breeders can better predict the performance of varieties, thereby improving breeding efficiency and enabling more targeted development of high-quality grapevine cultivars. In this study, 35 Primitivo clones were analysed, including selected and certified clones that have been propagated over several years in Apulia. Genetic variability among the Primitivo clones was assessed through genotyping by sequencing. Using 38,387 filtered SNPs, pairwise identity-by-state (IBS) analysis demonstrated the uniqueness of the 35 clones (IBS < 0.75), indicating a high degree of variability among the samples. Genetic diversity analysis revealed three primary groups, which were differentiated based on geographic origin. The clones from Gioia del Colle were grouped into two distinct clusters, which aligns with the observed variation in grape-related traits. The fixation index (*F*_ST_ > 0.50) identified numerous loci putatively associated with stress responses and developmental traits, including genes involved in key plant biological processes, stress response regulation, and adaptation to environmental conditions such as glutamate receptors, auxin, and ethylene signalling.

## 1. Introduction

Italy is recognized as the foremost global wine producer after Spain and France, with approximately 710,000 hectares of vineyard surface area and a production of 38,291 million hectolitres of wine in 2023 [1].

Apulia is a renowned wine-growing region in both Italy and Europe, covering an area of 95,000 hectares with a production of 1,202,860 tonnes in 2023 [2]. The region’s extensive latitude along the Adriatic plain and the Apennine ridge, coupled with 800 km of coastline exposed to the Adriatic and Ionian Seas, creates a variety of microclimates ideal for viticulture. Additionally, the numerous migrations since ancient times have introduced diverse plant material to Apulia, contributing to the extraordinary genetic richness of its grape germplasm. Until a few decades ago, Apulian viticulture focused on producing large quantities of bulk and high alcoholic wines, used to reinforce other products and often sold as unbottled wines, despite the richness and peculiarity of autochthonous varieties. Recently, increasing awareness of the region’s rich varietal heritage in combination with the development of oenological techniques has led to the recognition of the potential of varieties like Primitivo, Negramaro, Bombino, Susumaniello, also thanks to the development of numerous small vineyards, where farmers successfully maintain quality, biodiversity, and tradition [3]. Efforts to valorise local grape varieties, also supported by EU funds, have contributed to the revival and flourishing of Apulian viticulture, making the region the national wine producer with the highest density of wineries [4]. The most outstanding example of this valorisation is the Primitivo grape variety. Once primarily used to increase the alcohol content, intensity of colour, and structure of weaker wines, Primitivo has now achieved significant success under the protected designation of origin (PDO) mark, specifically Primitivo di Manduria and Primitivo di Gioia del Colle. In 2021, these PDOs saw extraordinary sales of 23 million litres, with a market value of EUR 195 million [5].

Primitivo is used to produce wines that achieve the highest average alcohol strength, dry extract values, glycerol, and also, in some cases, high residual reducing sugars due to incomplete alcoholic fermentation [6]. These wines feature spicy and red-fruit flavours [7], a ruby-purple colour that could be very stable over time, and a low polymerization rate of anthocyanin pigments, which could limit their aging potential [8]. According to tradition, Primitivo was selected in the territory of Gioia del Colle in the province of Bari towards the end of the 18th century as an early-ripening clone [9,10]. The variety is also found in other countries, where it is known under synonyms such as the Montenegrin Kratosija, the Croatian Crljenak Kaštelanski, and the American Zinfandel [11,12,13,14,15]. Studies on the origin of the Primitivo variety have revealed that the variety has strong genetic relationships also with Austrian, Croatian, and Hungarian varieties, such as the autochthonous Dalmatian cultivar Plavac Mali of which Zinfandel is one of the parents [16]. Despite these genetic connections, a divergence between Primitivo and these other varieties has been demonstrated, likely due to the spatial and temporal separation of Primitivo from the others [16].

The current success of the Apulian Primitivo has emphasised its rich intra-varietal diversity, prompting exploration of the “zoning” concept in the production area. The concept of “zoning” in viticulture or “viticultural zoning”, also referred to as the characterisation of “terroir zoning”, refers to the process of identifying and delineating specific areas within a wine-growing region that shares peculiar homogeneous environmental and growing conditions. These conditions include factors such as soil type, climate, topography, germplasm, and cultivation techniques. The main goal of zoning is to match grape varieties and specific clones with the optimal environmental conditions for their growth, thereby maximizing the specificity, the quality, and consistency of the wine produced [17]. Interest in this concept dates back to the 1970s, when it was recognised that ecotypes within polyclonal populations could enhance the diversity and uniqueness of the wines’ olfactory and flavour profiles, as well as improve adaptability to specific growing conditions or wine style/characteristics.

In Apulia, a regional, clonal, and sanitary selection programme was launched to promote the selection and comparative evaluation of the best clones and their molecular characterisation for varietal identity and product traceability. This also included the “sanitation” of candidate clones affected by virus and virus-like diseases and the evaluation of oenological quality of clones with distinctive characteristics. After at least three years of evaluation of the phenology, agronomic, productive performances, and oenological traits in official comparison and registration fields for at least three vintages, the programme led to the homologation of eight clones included in Italian National Register of Grape Varieties. These clones now represent the foundation of the current production of Apulian Primitivo [18].

To further valorise the clonal diversity of Apulian Primitivo and fully exploit its potential, it is necessary to better characterise its rich clonal genetic diversity. A molecular approach is essential to provide unique fingerprints and reveal cases of synonymy or misidentification. Microsatellites (simple sequence repeats; SSRs) have been the most commonly used markers for varietal identification due to their stability, high reproducibility, and polymorphism [19,20]. These markers are also used as references in several grapevine databases: Vitis International Variety Catalogue, https://www.vivc.de (accessed on 25 June 2024); European Vitis Database, http://www.eu-vitis.de/index.php (accessed on 24 June 2024); Italian Vitis Database, https://vitisdb.it/ (accessed on 24 June 2024) [21,22,23]. Recently, whole genome sequencing of the PN40024 grapevine genome, along with its several updated versions [24,25] as well as the genome assemblies of various grape cultivars [26,27,28,29], have facilitated the development of numerous single nucleotide polymorphisms (SNP) sets for *Vitis* spp. [30,31,32]. Reduced-representation sequencing methods such as genotyping by sequencing (GBS) and double-digest RAD sequencing (ddRADseq) offer flexible and cost-effective strategies for providing in-depth insights into the genetic architecture of germplasm collections [33,34,35].

Genomic information, encompassing genetic diversity, structural variants, allele frequencies, and phylogenetic relationships, is increasingly being used to inform decisions at both the population and individual levels. Integrating genomics with traditional phenotypic methods accelerates the selection process, enabling breeders to more accurately predict the performance of grapevine varieties or clones. Additionally, the combination of robust genomic data with terroir zoning offers a powerful framework for personalizing viticultural practices [36,37,38].

Genomic insights allow for the selection of grape varieties best suited to the specific conditions of each defined zone, while also facilitating the identification of genetic variants that improve grape yield and quality, tailored to the unique soil and climate conditions of each region.

In this study, we present a genome scan aimed at (i) exploring the intra-genetic variability within Primitivo clones retrieved from different areas of the Apulian region by the high-throughput GBS strategy and (ii) identifying divergent loci as a proxy for future sustainable breeding strategies.

## 2. Results

### 2.1. Genetic Diversity of Primitivo Clones

**Figure 1:**
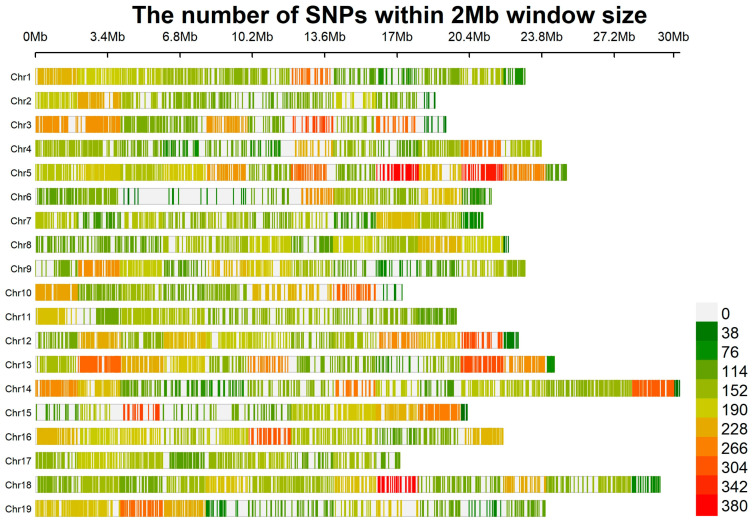
SNP density plot across the 19 chromosomes of *Vitis vinifera* L. illustrating the number of SNPs within a 2 Mb window size. The plot provides a detailed representation of SNP distribution, highlighting regions with varying SNP densities across the grapevine genome.

The analysed dataset consisted of 35 Primitivo clones and 184,895 unfiltered SNPs. After filtering, the number of SNP loci was reduced to 77,607, among which 38,387 polymorphic SNPs were identified in the Primitivo clones and retained for downstream analysis. The filtered VCF has been submitted in Mendeley Data, V2 (https://doi.org/10.17632/ktfz4nn959.2). Figure S1 reports the mean coverage depth per cultivar. Transitions (Ts) were more prevalent (63.7%) than transversions (Tv) (35.2%), resulting in a Ts/Tv ratio of 1.8. The most frequent substitution was C→T (31.9%), while the least frequent was C→G (6.8%) (see Table S2). Of the total SNPs identified, 18,997 (49.49%) were in genic regions, and 19,390 (50.51%) were in intergenic regions. Specifically, 23,108 SNPs were situated within annotated exons, impacting a total of 4820 genes. The distribution of SNPs across the chromosomes is detailed in Table S3 and Figure 1. Chromosomes 5 (2840 SNPs) had the highest number of SNPs, while chromosomes 6 (1311 SNPs) and 17 (2285 SNPs) had the fewest. The average SNP density across the chromosomes was ~1871.37 SNPs.

No duplicate Primitivo clones were detected in the dataset based on the pairwise IBS distance, with an average IBS distance of 0.75 between clones. Background levels of LD were reached at a distance of 11 kb (Figure S2).

**Figure 2:**
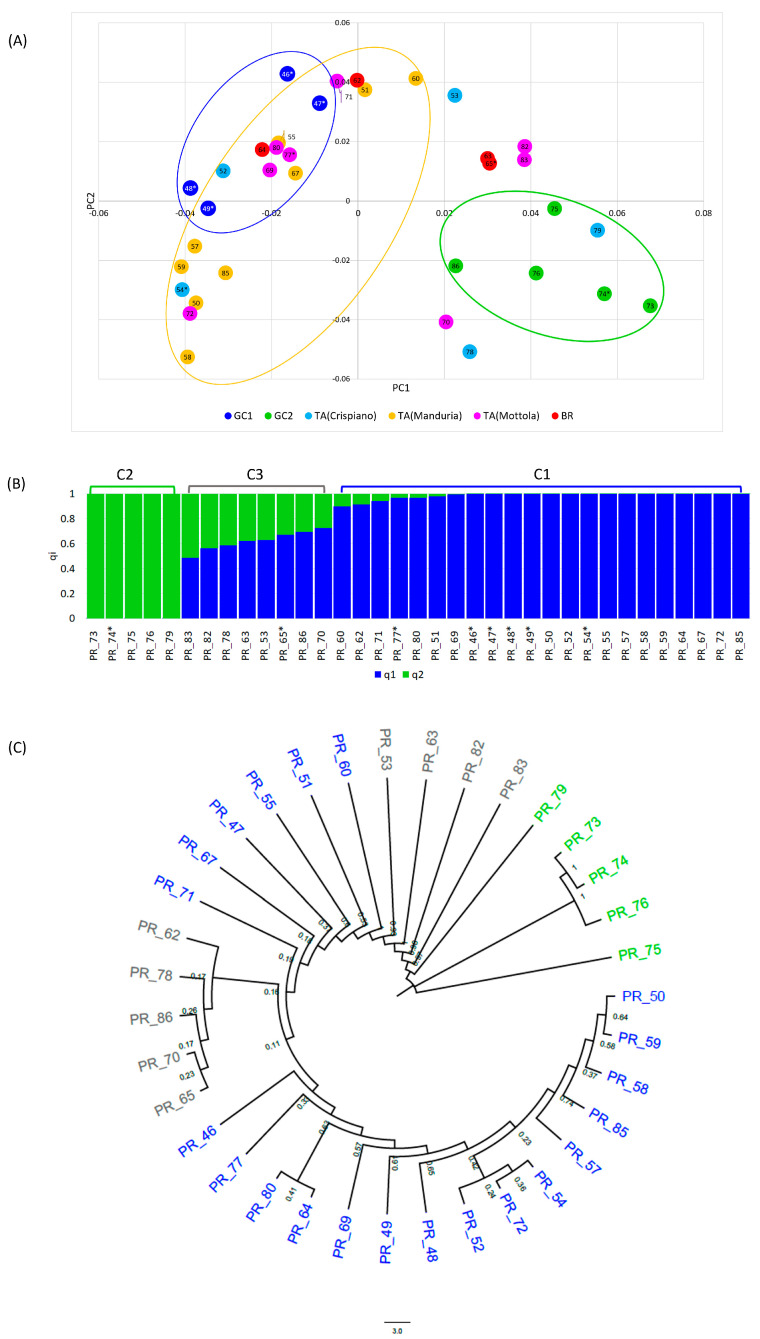
(**A**) A multidimensional scaling plot of the Primitivo clones. The plot visualizes the genetic relationships among the clones, with each point representing an individual clone. (**B**) A bar plot describing the population structure estimated by ADMIXTURE. Each bar is divided into K coloured segments, each representing the proportion of ancestry (qi) in each individual. (**C**) Neighbour-joining tree. The clusters were coloured based on ADMIXTURE sub-populations. Asterisks indicate registered clones.

Further data pruning was conducted to refine the dataset for population structure analysis, resulting in a final set of 22,908 SNPs. The MDS plot in Figure 2A displays the distribution of clones across two dimensions, revealing three main groups based on geographical origin. Clones from Gioia del Colle (GC) were in two distinct groups, GC1 and GC2, situated in the II and IV quadrants, respectively.

The GC1 cluster included only four registered clones, whereas the GC2 cluster comprised five clones, two of which were registered. The PC2 (y-axis) clearly distinguished clones from Manduria (a town in the province of Taranto), while the remaining clones from Taranto province were more dispersed throughout the plot.

Although the analysis of the population structure revealed that the optimal number into which to divide the population is K = 1, as expected in the case of clones, the stratification of the population at K = 2 was also evaluated (Figure S3). The two sub-populations identified by ADMIXTURE confirmed the separation of the GC2 clones into a distinctive sub-population labelled C2 (q2 = 1). Twenty-two clones were classified into the C1 sub-population (q1 > 0.90), which included individuals form various geographic origins. Eight clones were identified as admixed (q_i_ < 0.72), and from now on, we refer to this admixed group as C3 (Figure 2B). None originated from GC1 or Manduria. The neighbour-joining tree supported these findings, clustering clones PR_76, PR_74, and PR_73 into a separated clade, which was closely related to PR_75 and PR_79, all of which are grouped within GC2 (Figure 2C).

### 2.2. Divergent Loci and Putative Candidate Genes

Based on the panel of 38,387 SNPs, divergent loci between the Primitivo groups classified by ADMIXTURE (C1, C2 and C3) were identified. 

**Figure 3:**
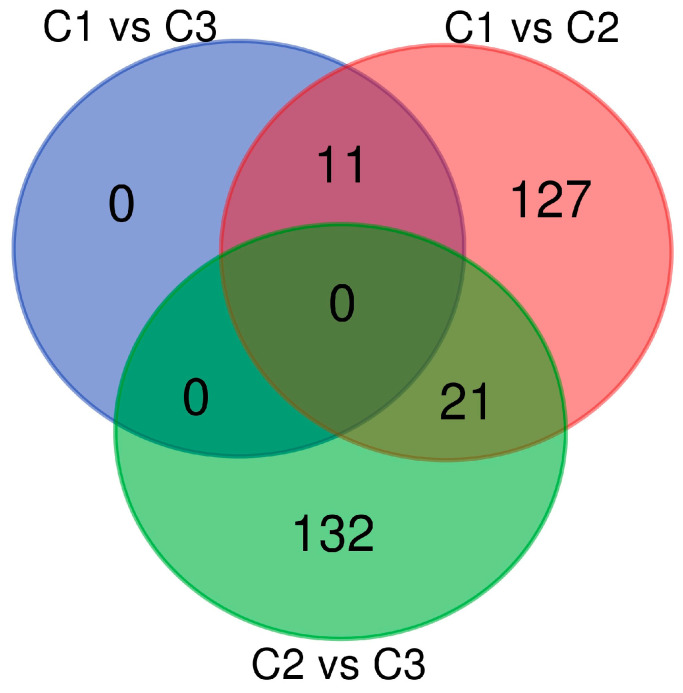
The Venn diagram illustrates the overlap of divergent loci in the three pairwise comparisons. The diagram visually represents the common and unique loci identified in each comparison, highlighting the genetic similarities and differences between the groups. The intersections indicate the number of shared loci, while the non-overlapping sections represent loci unique to each pairwise comparison.

The pairwise fixation index was calculated for individual SNPs, with a significance threshold set at >0.50. The analysis significance threshold revealed 11, 153, and 159 non-redundant SNPs for C1 vs. C3, C2 vs. C3, and C1 vs. C2 comparisons, respectively (Figure 3, Table S4). Among these, 43 and 29 SNPs fell within genes in C2 vs. C3 and C1 vs. C2 (Table S5).

All eleven divergent SNPs identified in C1 vs. C3 were identified also in the C1 vs. C2 comparison, while twenty-one were shared between C1 vs. C2 and C2 vs. C3. The eleven SNPs detected in C1 vs. C3 had *F*_ST_ values between 0.50 and 0.59, and were distributed only on chromosomes 4, 8, and 10. The fixation index (*F*_ST_)values in C1 vs. C2 and in C2 vs. C3 were higher, with maximum values of 0.94 and 0.89, respectively, and were distributed on all chromosomes with the exception of chr.5 in C1 vs. C2.

Due to the fairly rapid decay of LD, we chose to define a 50 kb region both downstream and upstream of the divergent SNPs to identify candidate genes. Seven SNPs detected on chr.4 in C1 vs. C3 and C1 vs. C2 flanked a cluster of *GLUTAMATE RECEPTOR* genes (VIT_204s0069g00295, VIT_204s0069g00320 and VIT_204s0069g00330), involved in responses to low temperature and salt stress in grapes, highlighting the clear differentiation between C1 and C2/C3.

Numerous signatures of divergence were identified between C1 vs. C2 (Table 1). Genes associated with divergent loci include those involved in plant adaptation to biotic and abiotic stress conditions and plant architecture. Remarkable genes are *NAC DOMAIN IPR003441* (VIT_202s0025g02710), *HISTONE ACETYLTRANSFERASE* (*HAT*, VIT_212s0034g00573), *BRANCHLESS TRICHOME* (*BLT*, VIT_202s0025g02670), *HYDROXYPYRUVATE REDUCTASE* (*HPR*, VIT_203s0038g02510), *BIREFRINGENCE-LIKE* (*TBL*, VIT_214s0066g01710), *WAX2-LIKE* (VIT_215s0045g01520, VIT_215s0045g01590, VIT_215s0045g01600), which are located on chromosomes 2, 3, 12, 14, 15, and 19, respectively.

The divergent SNPs were found to be significantly divergent in the C3 vs. C2 comparison, i.e., located in the genes *AUXIN-INDUCED 5NG4* (VIT_207s0005g03080), *PHYTOCHROME A* (VIT_214s0060g00100), *VAMP-LIKE YKT61* (VIT_214s0060g00090), *FASCICLINLIKE ARABINOGALACTAN* (VIT_214s0068g02005), *ETHYLENE-RESPONSIVE ELEMENT BINDING FACTOR* (VIT_214s0081g00520), *KTEL MOTIF-CONTAINING* (VIT_215s0046g03060), and *TRICHOME BIREFRINGENCE* (*TBL*, VIT_214s0066g01710).

Many genes were detected in both C1 vs. C2 and C3 vs. C2 comparisons such as *BIFUNCTIONAL NITRILASE NITRILE HYDRATASE NIT4B* (VIT_202s0109g00430), *RADICAL SAM* (SAM, VIT_201s0026g00050), *FRUCTOSE-BISPHOSPHATE ALDOLASE* (FBA, VIT_219s0015g01720), *URACIL PHOSPHORIBOSYLTRANSFERASE* (*UPRT*, VIT_219s0015g01730), highlighting the differentiation between C2 and the other two groups.

Finally, we identified another *TRICHOME BIREFRINGENCE* (*TBL*, VIT_201s0026g00810) located 130 kb from the divergent SNP on chromosome 1. Additionally, we considered the *TOBACCO MOSAIC VIRUS RESISTANCE* gene (TMV, VIT_212s0034g00512), which is situated 110 kb from the divergent SNP with an *F*_ST_ value of 0.94 on chromosome12.

## 3. Discussion

In recent decades, rising temperatures, increased CO_2_ levels, and greater dryness in wine-producing regions, have significantly impacted grapevine physiology and biochemistry, leading to changes in berry quality [39]. Given the global wine industry’s substantial socio-economic importance, research on grapevine germplasm biodiversity has become increasingly critical. Enhancing genetic diversity is now seen as essential for developing superior grapevine varieties that are more adaptable to changing environmental conditions while maintaining high-quality [40].

In grapevines, spontaneous somatic mutations give rise to cultivars containing multiple clonal lines, each exhibiting slight genotypic and phenotypic differences from the original mother plant. This intra-varietal variability can be beneficial for identifying potentially useful alleles associated with key traits in *Vitis vinifera* [41].

The Primitivo grapevine variety has garnered attention since the 1970s due to its rich clonal diversity and the potential for applying the concept of “zoning”. This approach focuses on identifying optimal interactions between grapevine clones and their environment, providing valuable insights that can guide stakeholders in developing new adaptive strategies [42,43,44]. The long-standing cultivation of Primitivo, including farmer-to-farmer exchanges, has likely contributed to its diverse clonal population [13]. This diversity is evident in the variability observed across the 35 clones analysed in this study. 

This highlights the importance of preserving clonal diversity to identify the best-performing clones for optimizing yield and quality across various growing conditions. According to the International Organization of Vine and Wine, as outlined in resolutions OIV-VITI 424/2010 and OIV-VITI 564B-2019, evaluating intra-varietal diversity and implementing polyclonal selection are essential strategies for preserving global grapevine heritage. In recent years, grapevine genetic diversity has been extensively studied using various genetic markers, including RFLP, AFLP, SSR, SNV, and SNPs [45]. These methods have been particularly applied to key varieties including Pinot Noir [46,47,48,49], Chardonnay [26,50,51,52], and the American synonym of Primitivo, Zinfandel [43,53,54]. The primary goal of this work is to leverage genomic tools and techniques to optimize the evaluation of grapevine genetic resources, strengthen conservation strategies, and advance selection and breeding in viticulture.

We employed a GBS-based approach to identify a robust set of clonal genetic variants, enabling a detailed exploration of genomic variability within the Primitivo clones. The extensive dataset of SNP markers generated through GBS provided a comprehensive genetic analysis, uncovering genomic differences that might have been overlooked by other techniques. While the use of 18 K SNPs to detect clonal variations in the biotypes of Aglianico and Muscat of Alexandria cultivars was insufficient to distinguish biotypes within the same cultivar [41], our approach demonstrated greater sensitivity in revealing subtle clonal differences.

The combination of datasets generated by RADseq and GBS approaches has previously been applied to study clones of grape varieties in southern Italy. However, this analysis revealed a notable lower number of SNPs [35] compared to those identified in Primitivo using only the GBS approach. In contrast, the analysis of Muscat of Alexandria clones using ~40 k SNPs derived from GBS successfully discriminated between clones [55], reaffirming GBS as the most suitable reduced-representation approach for rapid, large-scale SNP genotyping. This is largely due to the abundance of SNPs in intergenic regions, which tend to harbour more polymorphisms than coding regions.

The population structure analysis revealed that the selected Primitivo clones could be categorized into two main clusters, irrespective of their geographical origin. Notably, Primitivo clones from the Gioia del Colle district were distinctly separated. This clustering likely reflects the significant environmental differences that impact clonal variation, as previously observed by Alba et al. [56] and Meneghetti et al. [57,58]. Additionally, the division of Gioia del Colle clones into two genetic groups is consistent with observed variation in bunch-related traits, such as bunch weight and berry weight.

This likely stems from earlier preferences for higher-yielding clones between 1992 and 1996, which were characterized by larger size and weight. However, agronomic and oenological preferences have evolved over time, driven by shifts in consumer tastes and wine consumption habits favouring higher quality over quantity. A thorough understanding of genetic diversity, along with insights into past and current selection targets and novel allelic variations in Primitivo clones, will facilitate the introduction of potentially useful allelic variation and improve the performance of Primitivo under changing environmental conditions. Allelic richness, a key indicator of genetic variation, is crucial and serves for identifying populations that should be conserved. For Primitivo clones, the availability of diverse allele variants offers valuable opportunities for developing new strategies for Primitivo conservation, optimizing clonal selection and propagation by leveraging somatic variation.

The fixation index was used to identify divergent loci between populations, as revealed by population structure analysis, and to explore associations with putative candidate genes involved in key plant biological processes, stress response regulation, and adaptation to environmental conditions [59].

Within the identified groups, *F*_ST_ highlighted several divergent loci, notably associated with putative candidate genes essential for stress response and adaptation to both biotic and abiotic challenges [59]. Group C1 was distinct from both C2 and C3 due to SNP variants located near a cluster of *GLUTAMATE RECEPTOR* genes, which play a role in modulating plant growth and development in response to abiotic stresses. This gene cluster, identified on chromosome 4, was also reported by Sun et al. [60] using a genome-wide approach. Transcriptomic analysis demonstrated that these genes are regulated during grape leaf aging and response to cold and salt stress in fruits. The significant divergence between C1 and C2, as well as between C2 and C3, appears to be primarily associated with genomic regions involved in environmental stress responses. The gene showing the highest *F*_ST_ in the C1 vs. C2 comparison was *TMV*, indicating that virus resistance may have been a driver in clonal selection by breeders and/or farmers. Indeed, since the 1960s, clonal selection has focused on propagating vines with specific desirable traits, particular those free from viruses [61,62] or visible symptoms of viral diseases. Although *TMV* is not a virus of concern for grapevines, this gene may still play a role in the host’s broader response to viral infections, potentially influencing the modulation of symptoms caused by such pathogens.

Another divergent locus with a high *F*_ST_ value is located near the gene annotated as *HAT*, which is crucial for various aspects of berry development and ripening, as well as for responses to day length and environmental stresses [63]. Recent research has highlighted the role of *HAT*s as epigenetic modulators in the plant’s response to environmental stresses, supporting adaptation to diverse conditions [64,65]. Additionally, we identified the *HYDROXYPYRUVATE REDUCTASE* (*HPR*), which is essential for maintaining fundamental plant functions and plays an important role in adaptation to various stress conditions [66,67,68]. The enzyme was upregulated in grapevines following the application of dry yeast extracts (*DYEs*), which enhanced aromatic and secondary metabolites in wine while reducing oxidative stress in berries [69]. Yang et al. [70] pointed out the importance of the putative HPR-encoding gene *AtHPR1* (AT1G68010) in Arabidopsis, noting its crucial role in stress response, including drought resistance [71], high light conditions, and the maintenance of reactive oxygen species (*ROS*) homeostasis [72]. Similar functions have been observed in soybeans [73,74], suggesting a potential interaction between the enzyme and phytohormones. Divergence was observed in several genes associated with trichome development, including *TRICHOME BIREFRINGENCE-LIKE* (*TBL*) and *BRANCHLESS TRICHOME* (*BLT*) genes. Trichomes act as a protective barrier against various threats such as herbivores, ultraviolet (UV) radiation, pathogens, and excessive transpiration [74]. Their morphology and density are crucial for mediating interactions between the plant and its environment [75]. Additionally, trichomes contribute to increased epidermal thickness and higher content of long-chain fatty acids compared to other epidermal cells, helping to reduce evaporation and regulate temperature [76]. The *TBL* gene family is involved in trichome development; for instance, in Arabidopsis, TBL44 is associated with powdery mildew resistance [77], while TBL3 plays a crucial role in xylan acetylation [78,79].

Moreover, mutations in TBL genes in Arabidopsis can lead to reduced xylem vessels, extremely slow plant growth [80], dwarfism, weak stems, and stunted development [81]. In cotton, Wang et al. [82], identified four *TBL* genes involved in fibre formation and development [83], with overexpression of *GhTBL34* enhancing resistance to Verticillium wilt [84,85]. In rice, *TBL1* and *TBL2* affect the plant’s response to rice blight disease [86]. Overall, these studies emphasize the important role of *TBL* genes in breeding programmes, particularly their regulatory function in trichome development. Additionally, the *BRANCHLESS TRICHOME* (*BLT*) gene regulates trichomes branching by affecting endoreduplication in trichome precursor cells [87]. In Arabidopsis, the *BLT* gene acts as a negative regulator of complex trichomes development, with loss-of-function mutations resulting in an increased number of complex trichomes. Another divergent gene between the C1 vs. C2 groups is *WAX2-LIKE*, which is associated with the production of cuticular waxes crucial for protecting plant aerial organs from biotic and abiotic stressors [88]. In cucumber, *CsWAX2*, a homolog of Arabidopsis *AtWAX2*, is highly expressed in the epidermis and endoplasmic reticulum and is induced by low temperatures, drought, salt stress, and *ABA*. Transgenic cucumber plants expressing CsWAX2 exhibited significant improvements in pollen viability and enhanced resistance to water loss and pathogens compared to wild-type plants [89,90].

We have identified *NAC* transcription factors that are essential for flowering and the ripening of climacteric fruits, including grapes [91]. Specifically, *VvNAC26* promotes ripening by increasing levels of ethylene and abscisic acid [92].

Notably, we detected the *NIT4B* gene on chromosome 2, located within a selective sweep region identified by Liang et al. [93] through the whole-genome resequencing of 472 Vitis accessions. This gene is essential for cyanide detoxification and nitrogen recovery from cyanogenic glycosides, thereby impacting both plant development and defence.

Finally, a group of genes related to auxin and ethylene signalling pathways, as well as photoperiod-dependent processes such as flowering, distinguish group C2 from C3. Auxin and ethylene, along with genes involved in their regulation (e.g., *VAMP*, *KTEL*) act as the main stimuli influencing berry development, as well as phytochrome-mediated light perception [94,95,96]. Berry size and ripening processes significantly impact wine quality and production, which in turn have substantial economic implications. This likely influenced the selection of these clones by growers in specific areas [97], as in the case of Apulia.

While the results obtained are promising, this work is biased by a methodological limitation that should be addressed when developing a clonal sequencing protocol. Although the GBS technique offers high sequencing coverage and robust variant calling, providing a strong foundation for reliable data, sequencing multiple biological replicates are essential for assessing the reproducibility and reliability of the sequencing process. Although clonal material is genetically identical, technical variations in library preparation, sequencing, or data analysis can introduce errors. Sequencing multiple replicates enables the assessment of the consistency of genotype calls and helps identify and correct potential errors that may occur during the experimental workflow. Additionally, while a single clonal sample can suffice for certain applications, including replicates enhances the statistical power of the analysis and increases confidence in the accuracy of genotype identification. We believe that the high coverage and rigorous quality control measures in place reduced the likelihood of significant errors in the sequencing data. However, we recognize that incorporating replicates could improve the robustness of the clonal identification protocol. Unfortunately, we did not include biological replicates in our study, and we recognize that this could pose a limitation. Soon, we plan to use Sanger sequencing of the relevant SNPs to confirm the robustness of the results obtained. This step will be crucial for validating our findings and ensuring the accuracy of the identified genetic markers. By performing Sanger sequencing, we aim to provide a more detailed and reliable confirmation of the SNPs of interest, which will further strengthen the foundation for future genomic studies and breeding applications.

## 4. Materials and Methods

### 4.1. Plant Material and Phenotyping

**Figure 4:**
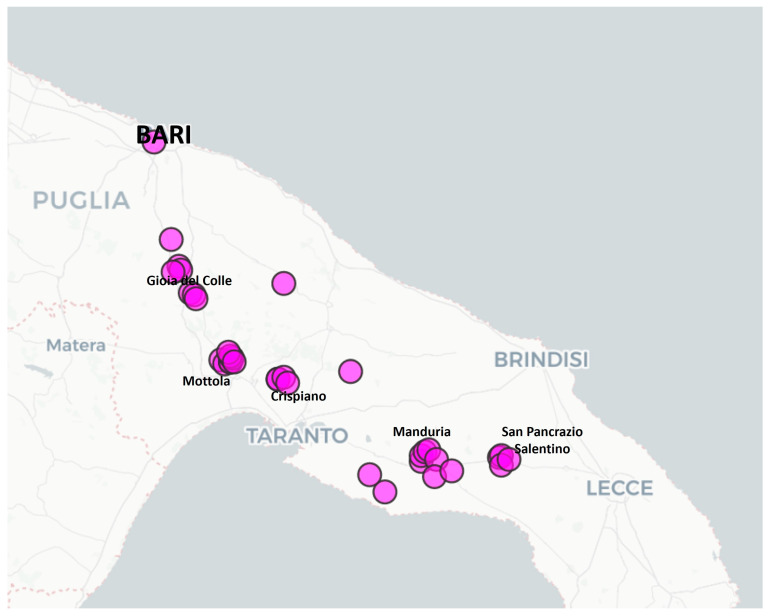
A map of Apulian region in southern Italy showing the distribution of Primitivo clones. Dot symbols are assigned based on geographical coordinates reported in Table S1.

The germplasm under investigation was conserved in a well-established regional ex situ gene bank managed by CRSFA-Centro Ricerca, Sperimentazione e Formazione in Agricoltura “Basile Caramia” in Locorotondo (Bari) [98] with a planting density of 1.0 × 2.5 m, following standard agronomic practices. The 35 analysed clones originated from various geographical locations in Apulia: 9 from the province of Bari, 22 from Taranto, and 4 from Brindisi. The clones from Taranto were further categorized based on their proximity to three towns, Mottola, Crispiano, and Manduria, which are ~74 km apart (Figure 4). The clone identifiers, sampling sites, provinces of origin, and geographical coordinates (latitude and longitude) are listed in Table 2. All samples were confirmed to be clones of the PRIMITIVO variety using a set of SSR markers routinely employed for grapevine genotyping: VVMD5, VVMD7, VVMD27, VVMD31, VVMD32, VVMS2, VRZAG62, and VRZAG79.

**Figure 5:**
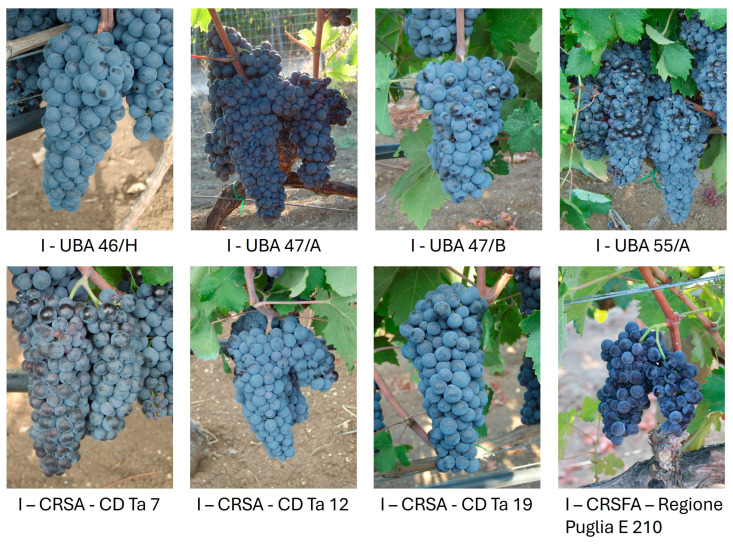
Bunches of the registered Primitivo clones in the Italian Grapevine Varieties Catalogue.

The clonal selection of the Primitivo cultivar began in the early 1970s and continued until 1995 across various areas of the Apulia region. This process took place in vineyards with plants of varying ages (ranging from a minimum of 35 years to over 60 years old) and featuring diverse training systems (“alberello”, trellis, or “spalliera” and “tendone” system). Over the past 15 years, among all the Primitivo selections conserved in the gene bank and tested by ELISA for 7 viruses (Grapevine Virus A (GVA), Grapevine Virus B (GVB), Grapevine Leafroll associated Virus 1 (GLRaV-1), GLRaV-2, GLRaV-3, Grapevine Fanleaf Virus (GFLV), and Grapevine Fleck Virus (GFkV), 35 promising clones were selected based on their distinctive phenotypic, the main productive characteristics, and the absence of key viruses that significantly affect critical quantitative production parameters. The phenotypic data summarized in Table S1 represent averages of measurements collected for these clones over several years. The clones were transferred to a conservation field at different times and monitored for the key production traits, including real fertility (number of bunches per plant/number of buds, yield (kg/vine), number of bunches per plant, bunch weight (g), berry weight (g), pruning wood weight (kg/vine), and the Ravaz index (yield/pruning weight). Real fertility, bunch weight, and berry size/weight were specifically evaluated due to their significant impact on overall yield, grape quality, and bunch compactness, which affects the susceptibility of grape clusters to rot. These data were used solely to illustrate the phenotypic variability that formed the basis for selecting the 35 clones in this study analysed at the genomic level. Among the samples, 8 clones were subsequently registered over time as follows: one in 1992, three 1996, three in 2013, and one in 2020. The clones resulted infected were submitted to sanitation treatments by meristem tip culture eventually associated with thermotherapy on in vitro explants. After the sanitation, these eight clones, according with the EU and Italian Certification schemes and protocols, were assessed for the absence of the 7 above-mentioned viruses plus the Arabis Mosaic Virus (ArMV) by RT-PCR as well the lack of the main symptoms of viral diseases (infectious degeneration, leafroll, rugose wood, rupestris stem pitting, Kober stem grooving, corky bark, vein necrosis, fleck) was proved by graft indexing on 5 woody indicators. Finally, the healthy vines underwent the necessary testing for registration in the Grapevine Italian National Catalogue in accordance with Ministerial Decree n. 489243 30 September 2021 (Figure 5 and Table S1) [99,100].

### 4.2. DNA Extraction, SNP Calling, Filtering, and Statistics

The methods described in Spadoni et al. [101] were utilized to extract and evaluate the quality and quantity of the gDNA. Elshire Group Ltd. (Palmerston North, New Zealand) performed GBS and SNP calling on 188 grapevine samples using the 12X.v2 version of the *Vitis vinifera* PN40024 reference genome sequence (assembly GCA_000003745.2) in 2017, following the methodology described by Miazzi et al. [31]. The resulting raw VCF file included data from 188 grapevine accessions and contained 184,895 unfiltered SNPs. Among these, half are Apulian, national, and international grapevine accessions, while the other half consists of clones of Apulian varieties, including 35 Primitivo clones. Some of these accessions were analysed in Miazzi et al. [31] and Villano et al. [35], while the remaining data are unpublished. For this study, 35 Primitivo clones were extracted from the original VCF file and then subjected to a filtering procedure using Golden Helix SNP and Variation Suite (SVS) v.8.8.4 (Golden Helix Inc., Bozeman, MT, USA) with the following parameters: minor allele frequency (MAF) < 5% and call rate > 20%.

VCFtools vs. 0.1.17 [102] was used to generate various statistics for the dataset under investigation and to add gene annotations to the VCF file. SNP density for each chromosome was visualized using the Circle Manhattan Plot (CMplot) R package 4.5.1 [103]. The dataset was LD (linkage disequilibrium)-pruned (r^2^ = 0.20) for population structure analysis using SVS.

### 4.3. Identity-by-State and Linkage Disequilibrium Analysis

The filtering procedure produced a high-quality SNP marker dataset, which was used to calculate the pairwise identical-by-state (IBS) distance matrix among all clones using SVS. Duplicated clones with an allele sharing rate ≥ 99% were considered duplicates, and only one of them was selected for downstream analysis. The Linkage Disequilibrium (LD) Adjacent Pairs Analysis function in SVS was employed to compute the average LD decay, predicted at the critical level of r^2^ = 0.20.

### 4.4. Genetic Diversity and Population Structure

Genetic diversity was evaluated using multidimensional scaling (MDS) using TASSEL v.5 [104], and a neighbour-joining tree with 1000 bootstrap replicates generated using MEGA v.11 [105]. The resulting tree was visualized with FigTree v.1.4 [106].

In this study, we used ADMIXTURE v. 1.3.0 [107] to identify potential gene pools within the Primitivo clones. Although population analysis by ADMIXTURE is typically employed for population structure analysis in large SNP datasets of unrelated individuals, we opted to use it as a preliminary exploratory tool to investigate the overall genetic structure. This analysis provided an initial framework for understanding genetic differentiation, which was then compared with more appropriate methods, such as neighbour-joining tree and PCA, both of which account for relatedness among individuals.

We performed 10-fold cross-validation (CV) for sub-populations (K) ranging from K = 1 to 10 with 1000 bootstrap replicates. The optimal K value was determined based on CV scores. Individuals were assigned to a specific group if their membership coefficient (q_i_) exceeded 0.90.

### 4.5. Divergent Loci Between Groups

To assess genomic divergence at individual loci between defined groups, we used the population-based pairwise *F*_ST_ index [108], as detailed in Taranto et al. [109]. Divergent loci with an *F*_ST_ ≥ 0.50 were considered for intra-clonal analysis. Candidate genes were identified using grapevine gene annotations from the source [110]. The LD decay distance was employed to define the region around each outlier marker, thus encompassing potential QTLs or genes associated with agronomically important traits.

## 5. Conclusions

This study highlights the substantial clonal diversity within the Primitivo grapevine variety, offering important insights for viticulture, especially in the context of climate change and shifting industry priorities. By employing genomic tools, particularly the GBS-based approach, we identified key clonal variations that could impact crucial traits such as yield, berry quality, and stress resilience. Genetic analysis revealed distinct clusters of Primitivo clones, closely associated with environmental factors and historical selection practices. These findings underscore the potential of genomic diversity to guide the selection of clones better suited to specific growing conditions, thereby improving both environmental sustainability and grape quality.

The study further demonstrates that genomic techniques, which facilitate a comprehensive exploration and utilization of intra-clonal genetic diversity, can greatly enhance the conservation, propagation, and effective use of grapevine germplasm. These advancements are particularly relevant for promoting climate-smart viticulture in Apulia. The genomic insights highlight the unique potential of each clone and provide valuable guidance for shaping future conservation and selection strategies for Primitivo clones, with far-reaching implications for the advancement of grapevine cultivation and genetic improvement.

Moreover, by integrating environmental data with genomic and future metabolomic analyses, we expect to gain a more comprehensive understanding of the molecular mechanisms driving the effects of terroir.

While the findings of this study are promising, several methodological limitations—such as the need for biological replicates to enhance sequencing robustness—should be addressed in future research to refine clonal sequencing protocols and further validate the results.

## Figures and Tables

**Table 1:** List of divergent SNP markers (*F*_ST_ > 0.50) associated with genes in Primitivo clones. In grey are SNPs located within genes.

Chr	Gene		Gene ID	Gene Description	Population Comparison	SNP ID
	Start	End				
1	8,677,792	8,680,892	VIT_201s0026g00050	*RADICAL SAM (SAM)*	C1_vs_C2, C3_vs_C2	1:8,680,396
1	9,655,509	9,657,926	VIT_201s0026g00810	*TRICHOME BIREFRINGENCE (TBL)*	C3_vs_C2	1:9,526,119; 1:9,526,127
2	2,326,977	2,327,833	VIT_202s0025g02670	*BRANCHLESS TRICHOME (BLT)*	C1_vs_C2	2:2,332,434; 2:2,332,445; 2:2,332,446; 2:2,332,452
2	2,347,183	2,348,922	VIT_202s0025g02710	*NAC DOMAIN IPR003441*	C1_vs_C2	2:2,332,434; 2:2,332,445; 2:2,332,446; 2:2,332,452
2	13,235,212	13,237,896	VIT_202s0109g00430	*BIFUNCTIONAL NITRILASE NITRILE HYDRATASE (NIT4B)*	C1_vs_C2, C3_vs_C2	2:13,240,982
3	1,749,439	1,753,048	VIT_203s0038g02510	*HYDROXYPYRUVATE REDUCTASE (HPR)*	C1_vs_C2	3:1,750,037
4	8,211,269	8,212,587	VIT_204s0069g00295	*GLUTAMATE RECEPTOR*	C1_vs_C2, C1_vs_C3	4:8,231,740; 4:8,231,751;
4	8,254,697	8,256,154	VIT_204s0069g00320			4:8,231,760; 4:8,231,768;
4	8,256,197	8,269,048	VIT_204s0069g00330			4:8,231,792; 4:8,231,812; 4:8,231,828
7	5,910,664	5,913,487	VIT_207s0005g03080	*AUXIN-INDUCED 5NG4*	C3_vs_C2	7:5,892,591; 7:5,898,409; 7:5,920,514
12	16,109,720	16,110,902	VIT_212s0034g00512	*TOBACCO MOSAIC VIRUS (TMV)*	C1_vs_C2	12:16,221,921
12	16,251,921	16,252,399	VIT_212s0034g00573	*HISTONE ACETYLTRANSFERASE (HAT)*	C1_vs_C2	12:16,221,921
14	114,200	121,866	VIT_214s0060g00100	*PHYTOCHROME A*	C3_vs_C2	14:117,578; 14:117,584
14	8,660,502	8,661,017	VIT_214s0081g00520	*ETHYLENE-RESPONSIVE ELEMENT BINDING FACTOR-LIKE PROTEIN*	C3_vs_C2	14:8,625,549; 14:8,625,552; 14:8,625,580; 14:8,625,590; 14:8,625,591; 14:8,625,595
14	93,389	98,859	VIT_214s0060g00090	*VAMP-LIKE YKT61*	C3_vs_C2	14:117,578; 14:117,584
14	28,045,323	28,048,092	VIT_214s0066g01710	*TRICHOME BIREFRINGENCE (TBL)*	C3_vs_C2	14:28,052,743
14	25,652,092	25,653,312	VIT_214s0068g02005	*FASCICLINLIKE ARABINOGALACTAN*	C3_vs_C2	14:25,652,715
15	7,543,895	7,547,614	VIT_215s0045g01520	*WAX2 - LIKE*	C1_vs_C2	15:7,625,555
15	7,639,601	7,643,264	VIT_215s0045g01590			15:7,625,555
15	7,647,421	7,648,971	VIT_215s0045g01600			15:7,625,555
15	19,692,688	19,693,047	VIT_215s0046g03060	*KTEL MOTIF-CONTAINING*	C3_vs_C2	15:19,692,917; 15:19,692,985
19	10,399,653	10,401,970	VIT_219s0015g01720	*FRUCTOSE-BISPHOSPHATE ALDOLASE (FBA)*	C1_vs_C2, C3_vs_C2	19:10,385,096; 19:10,385,169; 19:10,385,179
19	10,402,253	10,419,777	VIT_219s0015g01730	*URACIL PHOSPHORIBOSYLTRANSFERASE (UPRT)*	C1_vs_C2, C3_vs_C2	19:10,385,096; 19:10,385,169; 19:10,385,179

**Table 2:** List of Primitivo clones under study. For each clone, the identifier, sampling site, and province are reported. The national varietal register code for the registered/certified clones (marked with an asterisk) is provided in brackets.

Clone Identifier	Sampling Site	Province	Clone Identifier	Sampling Site	Province
PR_46 * (UBA 55/A)	Gioia del Colle	BA	PR_67	Manduria	TA
PR_47 * (UBA 46/H)	Gioia del Colle	BA	PR_69	Mottola	TA
PR_48 * (UBA 47/A)	Gioia del Colle	BA	PR_70	Mottola	TA
PR_49 * (UBA 47/B)	Bari	BA	PR_71	Mottola	TA
PR_50	Torricella	TA	PR_72	Mottola	TA
PR_51	Lizzano	TA	PR_73	Acquaviva delle Fonti	BA
PR_52	Crispiano	TA	PR_74 * (CD Ta 7)	Gioia del Colle	BA
PR_53	Crispiano	TA	PR_75	Gioia del Colle	BA
PR_54 * (CD Ta 19)	Crispiano	TA	PR_76	Gioia del Colle	BA
PR_55	Manduria	TA	PR_77 * (CD Ta 12)	Mottola	TA
PR_57	Manduria	TA	PR_78	Crispiano	TA
PR_58	Manduria	TA	PR_79	Crispiano	TA
PR_59	Manduria	TA	PR_80	Mottola	TA
PR_60	Manduria	TA	PR_82	Mottola	TA
PR_62	San Pancrazio Salentino	BR	PR_83	Mottola	TA
PR_63	San Pancrazio Salentino	BR	PR_85	Manduria	TA
PR_64	San Pancrazio Salentino	BR	PR_86	Alberobello	BA
PR_65 * (E 210)	San Pancrazio Salentino	BR			

## Data Availability

The filtered VCF file (35 Primitivo clones and 38,387 SNPs) has been uploaded to the Mendeley data repository under the title “Primitivo_clones” (Mendeley Data, V2, https://doi.org/10.17632/ktfz4nn959.2).

## Supplementary Materials

The following supporting information can be downloaded at https://www.mdpi.com/article/10.3390/plants14030437/s1. Figure S1: Bar charts showing mean depth per the 188 grapevines, including the 35 clones of Primitivo; Figure S2: Scatter plot showing the decay of linkage disequilibrium (r^2^ = 0.20) calculated for Primitivo clone germplasm; Figure S3: Cross-validation error estimates for each value of K (i.e., number of sub-populations) tested; Table S1: List of clones under study. For each clone, the Italian National Register of Vine Varieties code, the internal code, the phenotypic data, the sampling site, province, latitude, and longitude are reported; Table S2: Number of polymorphic SNPs before and after filtering procedure and statistics of high-quality SNPs calculated for Primitivo dataset; Table S3: The distribution of SNPs along the 19 chromosomes and the unknown chromosome (20); Table S4: Divergent SNP loci detected by pairwise comparisons between the sub-populations C1, C2, and C3 using *F*_ST_ (>0.50). SNP name, physical position on PN40024.v2 (chromosome and bp), reference and alternate alleles and *F*_ST_ values are reported; Table S5: List of candidate genes flanking the divergent SNPs (*F*_ST_ ≥ 0.50) in Primitivo clones. In bold are SNPs that fall within gene regions.
