# Supplementary figures and images for: Genome Scan Analysis for Advancing Knowledge and Conservation Strategies of Primitivo Clones (Vitis vinifera L.)
Source: Plants (Basel). 2025 Feb 2;14(3):437. doi: 10.3390/plants14030437 (PMC11821234; doi:10.3390/plants14030437)

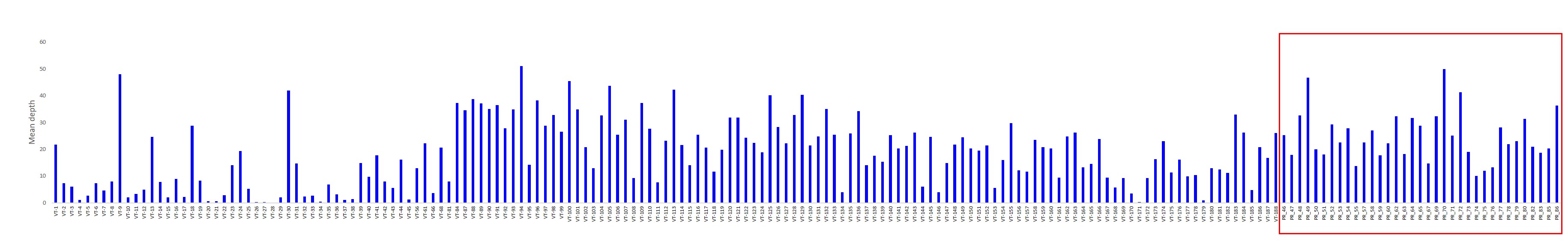

Supplement: Supplementary file 1 [file plants-14-00437-s001.zip › Supplementary Figure S1.jpg]

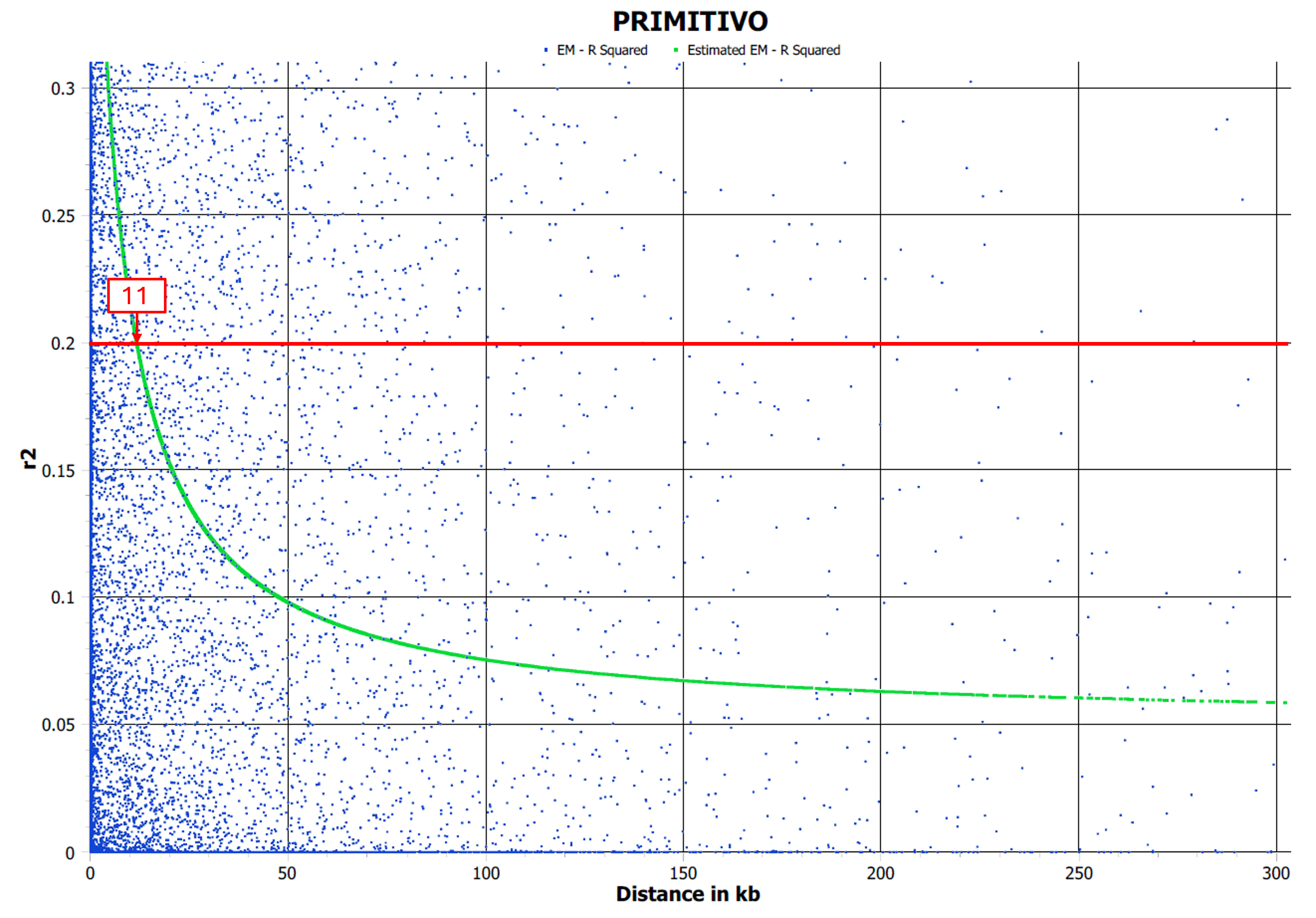

Supplement: Supplementary file 1 [file plants-14-00437-s001.zip › Supplementary Figure S2.jpeg]

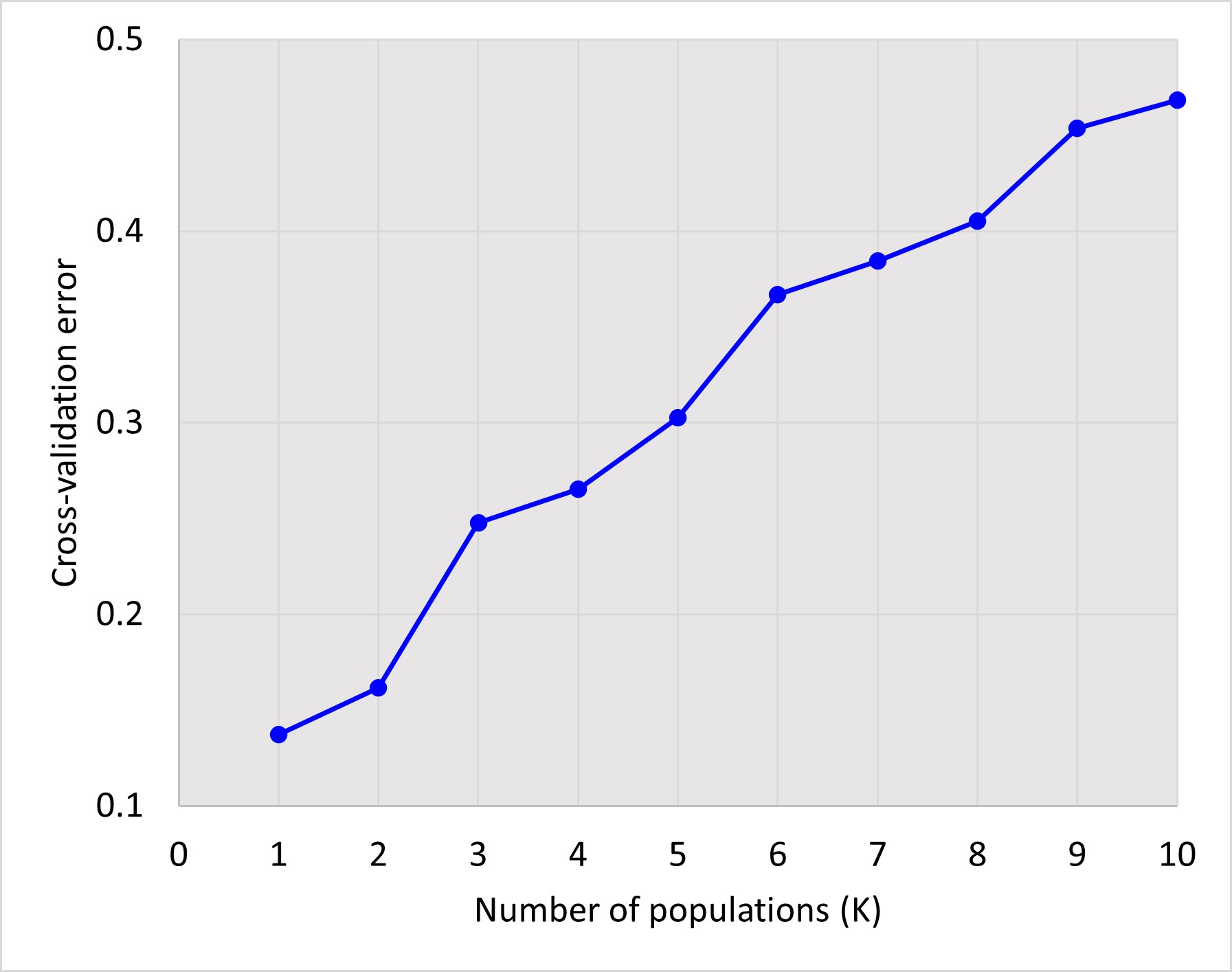

Supplement: Supplementary file 1 [file plants-14-00437-s001.zip › Supplementary Figure S3.jpeg]
